# Dietary Inflammatory Index and Cross-Sectional Associations with Inflammation, Muscle Mass and Function in Healthy Old Adults

**DOI:** 10.1007/s12603-022-1753-4

**Published:** 2022-02-24

**Authors:** U. Haß, C. Herpich, B. Kochlik, D. Weber, T. Grune, Kristina Norman

**Affiliations:** 1 University of Potsdam, Institute of Nutritional Science, Arthur-Scheunert-Allee 114-116, 14558, Nuthetal, Germany; 2 German Institute of Human Nutrition Potsdam-Rehbrücke, Department of Nutrition and Gerontology, Nuthetal, Germany; 3 German Institute of Human Nutrition Potsdam-Rehbrücke, Department of Molecular Toxicology, Nuthetal, Germany; 4 NutriAct-Competence Cluster Nutrition Research, Berlin-Potsdam, Germany; 5 German Center for Diabetes Research (DZD), Muenchen-Neuherberg, Germany; 6 German Center for Cardiovascular Research (DZHK), Partner Site Berlin, Berlin, Germany; 7 Department of Physiological Chemistry, Faculty of Chemistry, University of Vienna, Vienna, Austria; 8 Charité – Universitätsmedizin Berlin, corporate member of Freie Universität Berlin, Humboldt-Universität zu Berlin, Department of Geriatrics and Medical Gerontology, Berlin, Germany

**Keywords:** Inflammaging, healthy aging, nutrition, sarcopenia, physical function

## Abstract

**Importance:**

Inflammaging is considered a driver of age-related loss of muscle mass and function (sarcopenia). As nutrition might play a role in this process, the Dietary Inflammatory Index® (DII) has been developed to quantify the inflammatory potential of an individual diet.

**Objectives:**

We aimed to examine associations between the DII, inflammation, oxidative stress and sarcopenia-related parameters in healthy old compared to young adults.

**Design, Setting, and Participants:**

This cross-sectional study included data of 79 community-dwelling, healthy old adults (65–85 years) and 59 young adults (18–35 years) who participated in a randomized controlled trial from April to December 2019.

**Measurements:**

The DII was computed with dietary data collected from 24-h recall interviews. Associations between the DII, inflammatory and oxidative stress markers as well as bioimpedance-derived body composition, handgrip strength and gait speed were determined with multiple linear regression analyses adjusted for age, sex, physical activity and insulin resistance.

**Results:**

Regression analyses revealed significant relationships between a higher interleukin (IL) 6 and IL-6:IL-10-ratio and higher percentage fat mass (%FM), waist-to-height-ratio (WHtR) as well as lower percentage skeletal muscle mass (%SMM) and gait speed exclusively in old adults. Subsequent analyses showed that IL-6 was associated with a pro-inflammatory diet as indicated by a higher DII, again exclusively in old adults (beta coefficient (β)= 0.027, standard error (SE) 0.013, p=0.037). While the DII was not related with handgrip strength or oxidative stress in neither old nor young adults, linear models confirmed that a higher DII was inversely associated with gait speed in old participants (β= −0.022, SE 0.006, p<0.001). Finally, a pro-inflammatory diet was significantly associated with higher %FM, WHtR and lower %SMM in both age groups.

**Conclusion and Relevance:**

A pro-inflammatory diet reflected by the DII is associated with higher systemic inflammation, slower gait speed as well as lower muscle mass in old adults. Intervention studies are needed to examine whether anti-inflammatory dietary approaches can help to improve muscle mass and function and thus minimize the risk for sarcopenia in the long-term.

## Abbreviations

β: beta regression coefficient
BMI: body mass index
CRP: C-reactive protein
CV: coefficients of variability
DII: Dietary Inflammatory Index®
EDTA: ethylenediaminetetraacetic acid
ELISA: immunosorbent assays
FFQ: food frequency questionnaires
%FM: percentage fat mass
HOMA-IR: homeostasis model assessment — insulin resistance
IL: interleukin
MDA: malondialdehyde
SASP: senescence-associated secretory phenotype
SE: standard error
%SMM: percentage skeletal muscle mass
TNF-α: tumour necrosis factor alpha
WHtR: waist-to-height-ratio

## Introduction

**A**ccelerated aging is associated with a chronically inflamed phenotype and oxidative stress (1). Among other factors, the age-related increase in abdominal fat, immunosenescence and senescence-associated secretory phenotype (SASP) contribute to chronic, overactivated inflammatory reactions (inflammaging), thus promoting various age-related diseases (2, 3). Pro-inflammatory cytokines, such as tumour necrosis factor alpha (TNF-α), interleukin (IL) 1β or IL-6, play a dominant role in the underlying inflammatory processes (4).

Dietary patterns have been recognized to play an important role regarding inflammation; either having beneficial (Mediterranean diet) (5) or detrimental effects (Western diet) (6). In this context, the Dietary Inflammatory Index® (DII) was developed and validated globally within different cohorts to quantify the inflammatory potential of an individual diet (7, 8, 9, 10). The DII consists of 45 dietary parameters which have been associated with either pro- or anti-inflammatory effects on six of the most established inflammatory biomarkers (C-reactive protein (CRP), IL-1β, IL-4, IL-6, IL-10, TNF-α). Although the DII has been investigated in several pathologies such as cancer (11), depression (12) or cardiovascular disease (13), knowledge about associations between the dietary inflammatory burden reflected by the DII and sarcopenia-relevant outcomes in healthy old adults is still scarce. Since it is recognized that a chronic pro-inflammatory load contributes to loss of skeletal muscle and disability in higher age, this topic has gained more attention (14). It is well established, that sarcopenia development starts early in life (15). Therefore, the aim of this investigation was to examine associations between the DII and inflammaging as well as muscle mass and function in healthy old in comparison to young participants.

## Materials and methods

### Study design and population sample

This cross-sectional evaluation was performed in 80 healthy old (aged 65–85 years) and 60 healthy young adults (aged 18–35 years), who participated in a previously described study (16). The study was approved by the University of Potsdam ethics committee, registered at the German study register (DRKS00017090) and carried out in accordance with the Declaration of Helsinki. Exclusion criteria were any malignant or severe disease, any type of diabetes mellitus, stroke or heart attack within the last 6 months, food allergies or pregnancy. All participants gave written informed consent.

### Biomarker analyses

All blood samples were taken after an overnight fast between 8–9 am. Blood serum and EDTA plasma were stored at −80 °C until analysis.

#### Inflammatory and oxidative stress markers

To examine the inflammatory status, serum IL-6 [pg/mL] and IL-10 [pg/mL] were quantified using immunosorbent assays (ELISA) (IL-6 inter-assay coefficients of variability (CV): 4.7–5.0%, intra-assay CV: 4.2–5.1%; IL-10 inter-assay CV: 1.9–2.0%, intra-assay CV: 3.7–4.8%; BioVendor, Brno, Czech Republic). As a marker for oxidative stress, malondialdehyde (MDA) [*µ*mol/L] was measured in plasma samples by High-Performance Liquid Chromatography according to Wong et al. (17) with modifications by Weber et al. (18).

#### Metabolic parameters

Serum triglycerides [mmol/L] and serum glucose [mmol/L] were analysed using a colorimetric method (ABX Pentra 400, Horiba, Ltd. Japan). Serum insulin [*µ*U/mL] was quantified using ELISA (intra-assay CV: 4.8–6.0%, inter-assay CV: 8.1–9.0%; BioVendor, Brno, Czech Republic). The prevalence of insulin resistance risk was estimated using the homeostasis model assessment — insulin resistance (HOMA-IR) (19), where a value <2 is deemed normal, while an insulin resistance becomes very likely at values above 2.5 and values above 5 are typically found in persons with type 2 diabetes.

### Anthropometrics, muscle mass and function

Weight [kg], height [cm] and waist circumference [cm] were measured according to standard criteria, to subsequently calculate body mass index (BMI) [kg/m^2^] and waist-to-height-ratio (WHtR) as an indicator for abdominal obesity (>0.5) (20). Body composition was estimated with single-frequency bioelectrical impedance analysis (Bioimpedance Analyzer Quantum/S Akern Srl/RJL Systems, Florence, Italy) at 50 kHz with the participants lying in the supine position and electrodes placed on the right hand and foot. Skeletal muscle mass was calculated with the equation by Janssen et al. (21) and expressed in relation to body weight (%SMM). Percentage of fat mass (%FM) was calculated according to Kyle et al. (22). Maximum handgrip strength of the dominant hand [kg] was measured with a Jamar dynamometer (Sammons Preston Rolyan, Chicago, IL, USA). According to the standardized approach by Roberts et al. (23), participants were instructed to sit straight-backed with the feet placed flat on the floor, shoulder adducted but the elbow in 90° flexion, while forearm and wrist are in neutral position. Assessment of gait speed [m/s] was done during a 4-m walk test at the participants' usual pace in the old, but was not performed in the young participants due to its age specificity. Measures of muscle function were normalized to BMI, since body mass can influence physical performance (24).

### Physical activity

The International Physical Activity Questionnaire-short form was used to assess the time spent in intense, moderate or low activity within one week prior to the study (25).

### Dietary assessment and calculation of the DII

Dietary data were collected in a 24-h dietary recall interview. From initially 140 participants, 24-h recalls were available for 138 individuals. Dietary intakes were calculated with the nutrition software EBISpro version 2016 (Dr. J. Erhart, Willstätt-Legelshurst, Germany), which is based on the German Food Code and Nutrient Database (“Bundeslebensmittelschlüssel” version 3.02) with nearly 15.000 food items (26). Afterwards, computation of the DII was done in a stepwise manner following the instructions by Shivappa and colleagues (7).

### Statistics

Data analysis was performed in SPSS Statistics version 25 (IBM Corp., Chicago, IL, USA). The DII was analysed as continuous variable. Investigation of data distribution was done using Kolmogorov-Smirnov tests. According to the distribution, independent samples t-tests or Mann-Whitney U-tests were performed to compare continuous variables between the two age groups. Data are presented as either mean ± standard deviation or median with interquartile range. Differences in categorical variables were determined with Chi-square test. Associations between the DII and inflammation, oxidative stress levels, as well as muscle mass and function were assessed with multiple linear regression analyses. Additionally, these analyses were performed in a sub-sample within the old study group characterized as sedentary (n=59). In case of non-normal distribution, data has been log-transformed. Adjustments were made for age and sex (model 1), and additionally for physical activity since this is recognized to affect the inflammatory condition (model 2). Regression analyses regarding inflammatory and oxidative stress markers were further adjusted for insulin resistance (model 3), since there were differences between age groups. To allow for discrimination for the risk of insulin resistance, the cut-off for HOMA-IR was set at 2.5. Results are presented as beta regression coefficient (β) with standard error (SE). Statistical significance was assumed at p<0.05.

## Results

### Subject characteristics

79 old (72.4±5.5 years) and 59 young (26.5±4.2 years) participants were included in our analyses. Approximately 75% of the participants were female and sex was equally distributed in both groups. Key subject characteristics and relevant biomarker concentrations are shown in Table 1. Old participants showed significantly lower muscle mass and strength compared to young participants. Moreover, old adults had significantly higher inflammatory levels and significantly higher MDA concentrations compared to young adults. Additionally, old participants exhibited significantly higher triglyceride and glucose levels as well as higher HOMA indices compared to the young participants.

**Table 1:** Characteristics and biomarker concentrations of old and young participants

	**Young (n=59)**	**Old (n=79)**	**p-value#**
Sex [n]	♀ 43 / 16 ♂	♀ 59 / 20 ♂	0.811
Age [years]	26.1 (6.3)	72.4 (8.9)	—
Body mass index [kg/m^2^]	22.8 (3.6)	28.8 (6.2)	0.009
Waist-to-height-ratio	0.44 (0.05)	0.56 (0.11)	0.009
Fat mass [%]	29.0±6.2	34.0±6.9	0.009
Skeletal muscle mass [%]	37.3±5.2	30.5±5.6	0.009
Handgrip strength [kg]	36.2 (10.6)	28.5 (12.0)	0.009
Gait speed† [m/s]	—	1.35 (0.29)	—
Dietary Inflammatory Index	2.73 (2.56)	3.11 (3.15)	0.668
Triglycerides [mmol/L]	0.80 (0.47)	1.09 (0.62)	0.014
Glucose [mmol/L]	4.58 (0.39)	5.24 (0.56)	0.014
Insulin [*µ*U/mL]	10.2 (2.9)	10.2 (4.0)	1.0
HOMA-IR	2.1 (0.7)	2.35 (1.05)	0.014
IL-6 [pg/mL]	2.87 (1.66)	3.56 (2.30)	0.014
IL-10 [pg/mL]	5.34 (2.01)	7.64 (2.55)	0.014
IL-6:IL-10-ratio	0.46 (0.35)	0.46 (0.31)	1.0
MDA [*µ*mol/L]	0.57 (0.30)	0.86 (0.49)	0.014


Values are presented as mean ± standard deviation or median with (interquartile range) and p-value for significance level after independent samples t-Test or Mann-Whitney U-test and #Bonferroni correction to counteract type I error; sex distribution was tested with Chi-square; †measurement not performed in young participants; HOMA-IR homeostasis model assessment — insulin resistance; IL interleukin; MDA malondialdehyde

### Inflammatory status and associations with muscle strength, muscle function and body composition

Fully-adjusted regression analyses revealed significant relationships between a pro-inflammatory status as indicated by higher IL-6 and IL-6:IL-10-ratio and sarcopenia-related parameters as indicated by lower %SMM, handgrip strength and gait speed in the old group, but not in the young group (Table 2). Moreover, higher %FM and WHtR were also significantly associated with higher IL-6 and IL-6:IL-10-ratio.

**Table 2:** Associations between inflammatory status and body composition as well as muscle function in old and young participants

		**Young (n=59)**			**Old (n=79)**		
		**IL-6**	**IL-10**	**IL-6:IL-10**	**IL-6**	**IL-10**	**IL-6:IL-10**
WHtR	β	−0.009	−0.008	0.000	0.097***	0.021	0.079**
	SE	0.028	0.025	0.022	0.026	0.063	0.025
%FMǂ	β	−0.172	1.451	−1.206	9.770***	1.812	8.039**
	SE	3.318	3.029	2.633	2.575	6.148	2.432
%SMMǂ	β	−0.607	−2.508	1.511	−6.218**	−2.984	−4.787*
	SE	2.676	2.425	2.119	1.908	4.444	1.806
HGS/BMI	β	−0.079	0.010	−0.058	−0.154**	0.040	−0.139*
	SE	0.052	0.049	0.041	0.057	0.130	0.053
Gait speed/BMI†	β	—	—	—	−0.217***	0.014	−0.188***
	SE				0.048	0.120	0.046


Values are presented as β beta coefficient with SE standard error after multiple linear regression analysis with log-transformed data at baseline, adjusted for age, sex, physical activity and insulin resistance; significant associations marked as *p<0.05 **p<0.01 ***p<0.001; ǂnot log-transformed; †measurement not performed in young participants; %FM percentage fat mass; Gait speed/BMI gait speed normalized to body mass index; HGS/BMI handgrip strength normalized to body mass index; IL interleukin; %SMM percentage skeletal muscle mass; WHtR waist-to-height-ratio

### Dietary assessment and dietary inflammatory load

The mean dietary inflammatory load reflected by the DII did not differ between both groups (Table 1). Caloric and carbohydrate intake were significantly different between groups, whereas other macronutrient intakes were comparable (Supplemental figure 1 a and b).

### The DII and its associations with inflammation and oxidative stress

Subsequent multiple linear regression models revealed that higher DII scores were significantly associated with higher IL-6 concentrations in the old group, but not in the young group (Table 3). However, the DII was not associated with MDA concentrations in old and young adults (Table 3).

**Table 3:** Associations between the Dietary Inflammatory Index® and inflammatory status, oxidative stress, metabolic parameters, body composition and muscle function in old and young participants

	**Young (n=59)**			**Old (n=79)**		
	**β**	**SE**	**p-value**	**β**	**SE**	**p-value**
Inflammation						
IL-6	0.005	0.015	0.752	0.027	0.013	0.037
IL-10	0.021	0.016	0.200	0.008	0.006	0.161
IL-6:IL-10	−0.016	0.019	0.391	0.019	0.014	0.186
Oxidative stress						
MDA	−0.021	0.013	0.105	−0.019	0.011	0.085
Metabolic profile						
Triglycerides	0.021	0.013	0.108	0.031	0.010	0.002
Glucose	0.000	0.002	0.917	0.009	0.003	0.012
Insulin	0.016	0.007	0.037	0.028	0.009	0.002
HOMA-IR	0.016	0.008	0.053	0.036	0.010	0.001
Fat distribution						
WHtR	0.007	0.003	0.028	0.012	0.003	<0.001
%FM	0.915	0.338	0.009	0.744	0.310	0.019
Muscle mass and function						
%SMM	−0.709	0.271	0.011	−0.524	0.229	0.025
HGS/BMI	−0.010	0.005	0.078	−0.005	0.006	0.397
Gait speed/BMI †	—	—	—	−0.022	0.006	<0.001


Values are presented as β beta coefficient with SE standard error and p-value for significance level after multiple linear regression analysis with log-transformed data at baseline adjusted for age, sex, physical activity and regarding inflammatory/oxidative stress markers also for insulin resistance; †measurement not performed in young participants; %FM percentage fat mass; Gait speed/BMI gait speed normalized to body mass index; HGS/BMI handgrip strength normalized to body mass index; HOMA homeostasis model assessment — insulin resistance; IL interleukin; MDA malondialdehyde; %SMM percentage skeletal muscle mass; WHtR waist-to-height-ratio

### The DII and its associations with muscle strength, muscle function and body composition

As can be seen in Table 3, regression analyses adjusted for age, sex and physical activity showed that higher DII scores were not significantly associated with handgrip strength, but with lower gait speed and lower %SMM in old adults. In addition, significant associations were found between higher DII scores and higher %FM as well as WHtR in both age groups.

### The DII and its associations with metabolic status

The DII was significantly positive associated with triglyceride, glucose, and insulin concentrations, as well as HOMA indices in the old group, whereas significant positive associations were only seen between the DII and insulin concentrations in the young group (Table 3).

## Discussion

This cross-sectional analysis showed that a pro-inflammatory diet reflected by a higher DII score was associated with higher systemic inflammation and slower gait speed as well as poorer muscle mass in old adults. A higher DII was further associated with higher (abdominal) fat mass and metabolic markers.

In accordance with a typical German, westernized diet, the overall dietary intake was predominantly pro-inflammatory in both age groups (Table 1). This can be attributed to a relatively high fat intake, particularly a high intake of saturated fatty acids (Supplemental figure 1 b) and omega-6:omega-3-ratio (Supplemental figure 1 c). Omega-3 and omega-6 fatty acids are known for their modulating effects on inflammatory processes (27) and a dietary intake low in omega-6:omega-3-ratio of ideally ≤2:1 is associated with a reduced pro-inflammatory cytokine release (28). Furthermore, smoking (29), alcohol consumption (30), and medication use (31) can affect the inflammatory status. However, the number of smokers was low and alcohol intake between groups was comparable (data not shown). Young participants did not take any medication, but the medication taken by old participants might have had an effect on inflammation parameters, as for example statins are known for their anti-inflammatory effects (31).

The DII was positively associated with IL-6 in the old group but not in the young group, which might imply a better compensation of the pro-inflammatory dietary load in young adults. The observed association became even more prominent in a sub-sample characterized as sedentary old (Supplemental figure 2 a). This is plausible, as physical activity is associated with anti-inflammatory effects also at an older age (32). Moreover, higher DII scores were accompanied by significantly higher IL-6 but not by IL-10 in the old participants, which might imply a failing anti-inflammatory compensatory mechanism contributing to inflammaging (33).

Additionally, the associations between the DII and metabolic parameters within the old group might be interpreted as a limited metabolic response capacity to the higher inflammatory dietary load, also possibly as part of the inflammaging process (34). This also seems to be reflected by the observed unfavourable body composition including lower %SMM, higher %FM and higher WHtR in relation to the pro-inflammatory status.

Regarding muscle mass and function, the DII was negatively associated with %SMM and gait speed in the old group, but not with handgrip strength. Significant associations between higher DII and lower handgrip strength were only seen in the sedentary sub-sample (Supplemental figure 2 b), which might be attributed to the fact that the whole old study group still performed quite well in relevant functional parameters.

A recent retrospective analysis within the Australian Geelong Osteoporosis Study indicated that a long-term anti-inflammatory diet reflected by a lower DII is associated with higher muscle mass in men (35) and women (36). Chronic irregulated inflammatory processes in general are recognized as drivers for age-related diseases, including musculoskeletal disorders. Therefore, a diet with low inflammatory load possibly represents a relevant tool to prevent loss of muscle mass in aging by supporting cytokine balance.

### Strength and Limitations

Originally, the DII is computed based on food frequency questionnaires (FFQ). Contrarily, we used 24h-recall based data to calculate the DII, which does not reflect habitual diet as reliably. However, it has been recommended by the developers of the DII to use an open-ended strategy to gain more dietary information and thereby increase the chance to include most of the 45 DII-relevant dietary parameters (7). In the present study, 31 from 45 possible DII parameters were available for its calculation, which is higher than the average expected 25–30 DII parameters from FFQs (37). Beside IL-6 and IL-10, future studies might include additional inflammation markers, since the DII has also been associated with CRP, IL-1β, IL-4 and TNF-α. Certainly, one main limitation of our study is the small sample size. Nonetheless, our results are in agreement with other studies with more participants (38). Furthermore, BIA measurements must be interpreted with caution. In order to assess fat and fat free mass it might not be as accurate as dual x-ray absorptiometry; however, using adequate equations, BIA provides comparable estimates of skeletal muscle mass in healthy adults (39).

## Conclusion

We conclude that a pro-inflammatory diet is associated with higher systemic inflammation as well as adverse body composition expressed by higher fat mass including higher abdominal fat distribution in healthy community-dwelling old adults. More interestingly, a pro-inflammatory diet is also associated with poorer muscle mass and slower gait speed, both sarcopenia-relevant parameters, in old adults. Intervention studies are needed to examine whether anti-inflammatory dietary approaches can help to improve muscle mass and function and thus minimize the risk for sarcopenia in the long-term.

## Electronic Supplementary Material

Supplementary material, approximately 206 KB.
