# Supplementary material for: Dietary Inflammatory Index and Cross-Sectional Associations with Inflammation, Muscle Mass and Function in Healthy Old Adults
Source: J Nutr Health Aging. 2022 Feb 24;26(4):346–51. doi: 10.1007/s12603-022-1753-4 (PMC12879146; doi:10.1007/s12603-022-1753-4)
Supplement: Supplementary file 1 — Supplementary material, approximately 206 KB. [file mmc1.docx]

# Supplemental

**
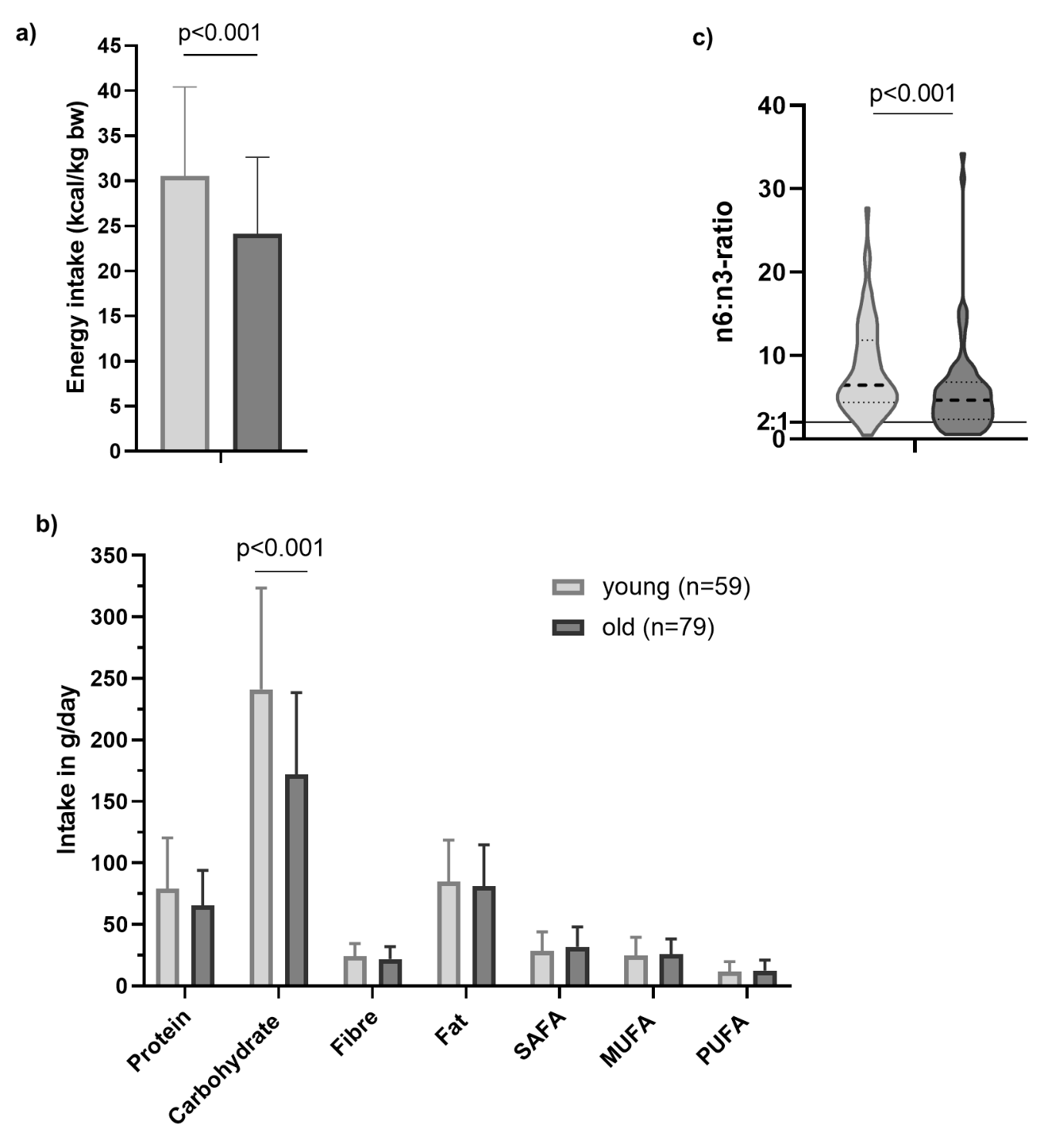
**

**Supplemental figure 1 Comparisons between old and young participants regarding a) energy intake in kilocalorie (kcal) per kilogram body weight (kg bw) per day, b) nutritional intake in gram (g) per day and c) distribution of daily omega (n)-6 to n-3 intakes with line at 2:1 indicating ideal intakes; MUFA** mono-unsaturated fatty acids**, PUFA** poly-unsaturated fatty acids**, SAFA** saturated fatty acids


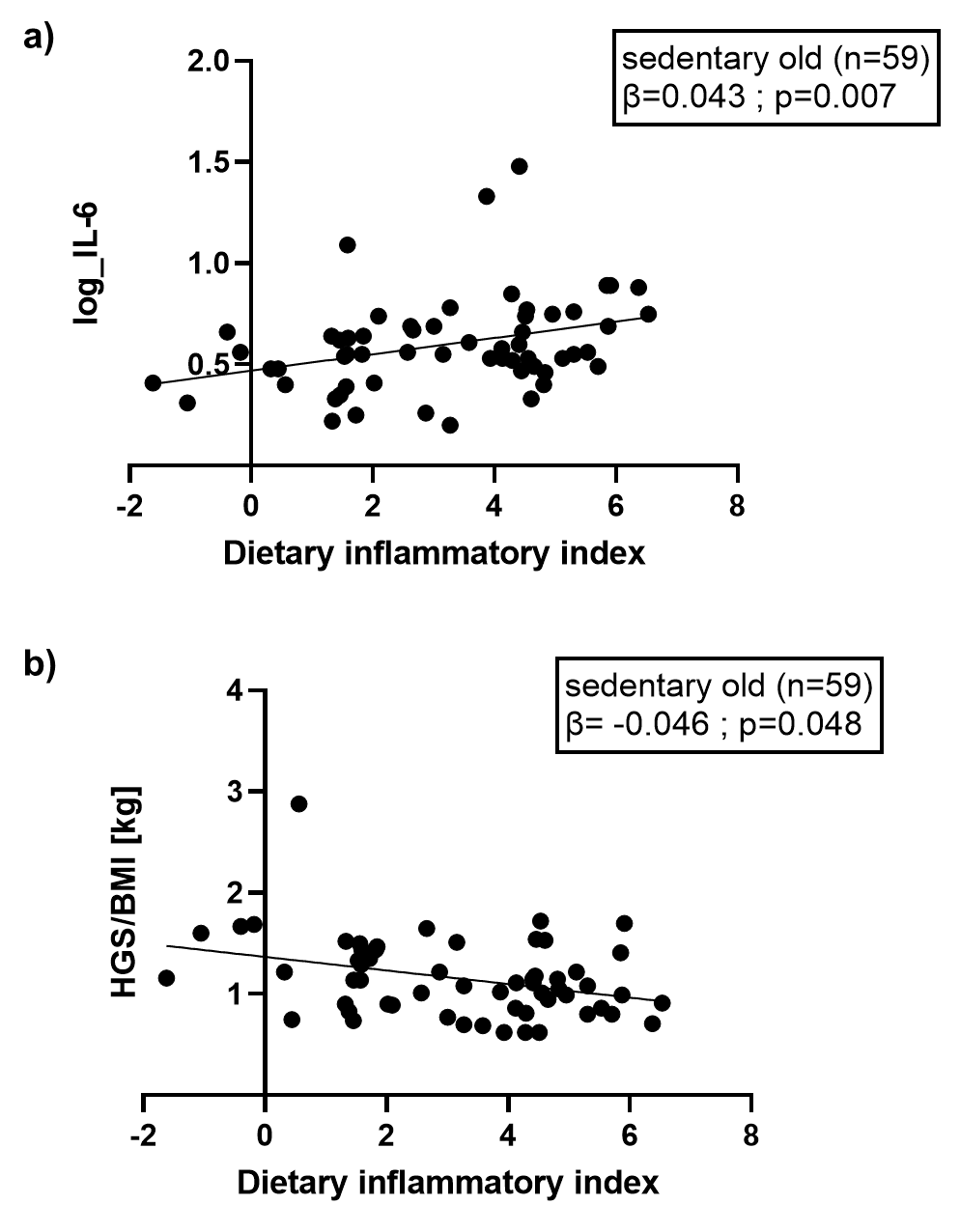


**Supplemental figure 2 Regression analysis adjusted for sex between dietary inflammatory index and a) log-transformed interleukin 6 (IL-6) and b) handgrip strength of the dominant hand normalized to body mass index (HGS/BMI) in a sub-sample characterized as sedentary old adults**
